# Complete mitochondrial genome of *Appasus japonicus* Vuillefroy, 1864 (Hemiptera: Belostomatidae)

**DOI:** 10.1080/23802359.2022.2152198

**Published:** 2022-12-11

**Authors:** Jung-Soo Han, Kyeong-Sik Cheon, Ji-Eun Jang, Jun-Kil Choi, Hwang-Goo Lee

**Affiliations:** Department of Biological Science, Sangji University, Wonju, South Korea

**Keywords:** *Appasus japonicus*, complete mitochondrial genome, phylogeny

## Abstract

We describe the initial sequencing and assembly of the complete mitochondrial genome of *Appasus japonicus* Vuillefroy, 1864 (Hemiptera; Belostomatidae; Appasus). The mitochondrial genome of *A. japonicus* was found to be 18,608 bp. It contains thirteen protein-coding genes (PCGs), 22 transfer RNAs (tRNAs), two ribosomal RNAs (rRNAs) and an AT-rich region. The overall base composition of *A. japonicus* is A-41.9%, C-17.5%, G-11.9%, and T-28.7%. A phylogenetic analysis of 21 species within the order Hemiptera suggests that *Diplonychus rusticus* is most closely related to *A. japonicus*.

## Introduction

**Figure 1.**
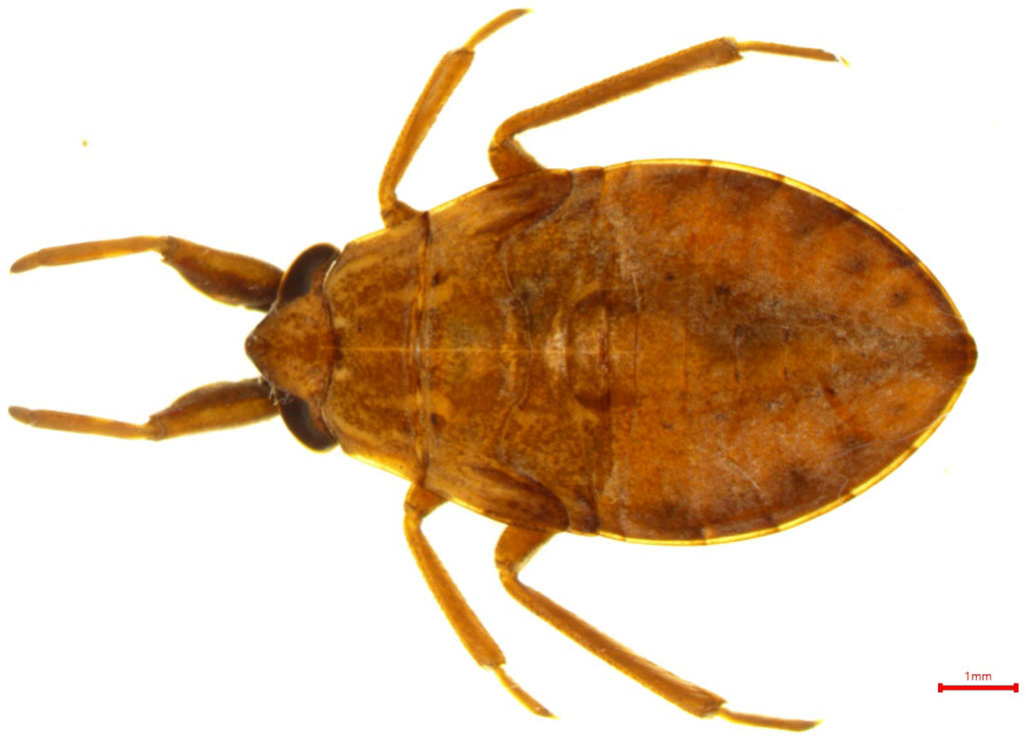
*Appasus japonicus* reference image.

Some hemiptera prefer slow-flowing environments and habitats rich in aquatic plants. These preferences also play a role in population regulation in freshwater ecosystems in terms of predator positions in the food chain (Mukai et al. 2005). *Appasus japonicus* Vullefroy 1864 (Hemiptera, Belostomatidae) had been classified as a genus of *Appasus* (Figure 1). In Korea, the family Belostomatidae includes *Diplonychus esakii, Lethocerus deyrollei, Appasus japonicus* and *Appasus major*. However, genetic research on Belostomatidae is lacking in Korea. For this reason, this study analyzed the complete mitochondrial genome of *Appasus japonicus*, which shows the widest distribution range in Korea among the species in Belostomatidae.

## Materials and methods

*A. japonicus* was collected from Nakwaamcheon, Buseok-myeon, Yeongju-si, Gyeongsangbuk-do (36°57'40.30"N, 128°39'34.76"E). The specimens were identified by Hwang-Goo Lee (morningdew@sangji.ac.kr) and then stored in the Department of Biological Science of Sangji University in Korea under voucher number SJAEAJ001. *A. japonicus* is an invertebrates and is neither an endangered or protected species, thus meeting the requirements for ethical approval.

**Figure 2.**
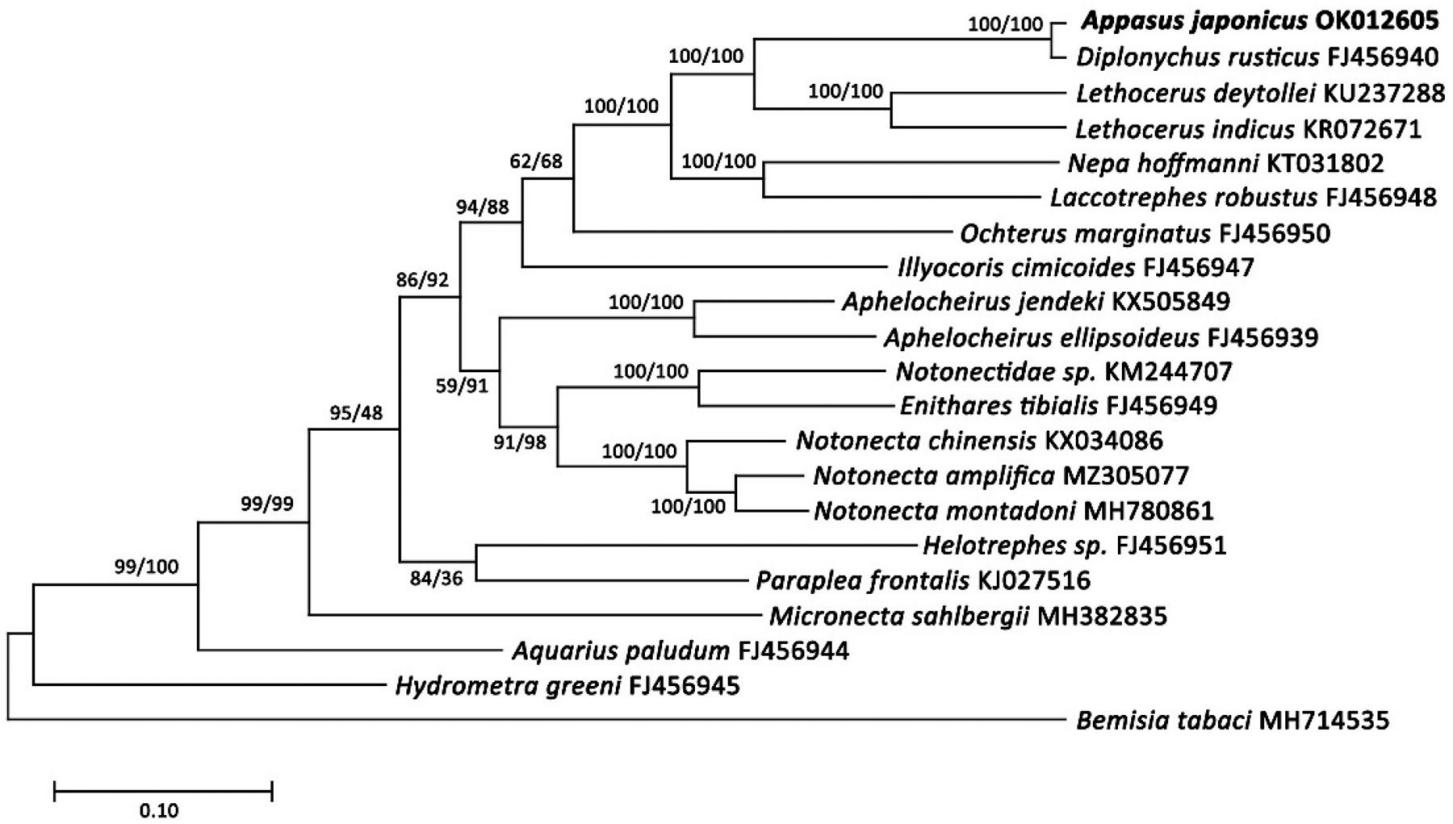
Phylogenetic tree of 21 species in the order Hemiptera including *A. japonicus*. Reconstrction of the maximum likelihood (ML) and neighbor joining (NJ) trees was based on 15 genes (13 PCGs, two rRNA). Numbers at the branches represent the bootstrap support values for ML (left) and NJ (right), respectively. Branching patterns and branch lengths follow the results of the ML analysis.

We also compared each gene to the previously published mitochondrial genome of *D. rusticus* (GenBank FJ456940), which was suggested to be the most closely related species (Choi et al. 2021), for correct gene annotation. To reveal the phylogenetic position of *A. japonica*, the species used in the mitochondrial genome paper of *D. rusticus* and Hemiptera species registered in Korea were downloaded. As outgroups, *Bemisia tabaci* was downloaded and a total of 20 species were used. Their GenBank registration numbers are shown in Figure 2. Phylogenetic analyses were done using thirteen PCGs and two rRNA genes from the 21 species. Total genomic DNA was extracted from the specimen using a DNeasy Blood & Tissue Kit (Qiagen, Hilden, Germany). Genome sequencing was performed on the MiSeq (Illumina Inc., San Diego, CA) platform. The treatments of the raw data, such as trimming, were performed with Geneious prime 2021.1.1 (Biomatters Ltd, Auckland, New Zealand). The sequences were aligned using MAFFT (Katoh et al. 2002) and the maximum-likelihood (ML) and neighbor-joining (NJ) trees were created with MEGA X (Kumar et al. 2018).

## Results

**Figure 3.**
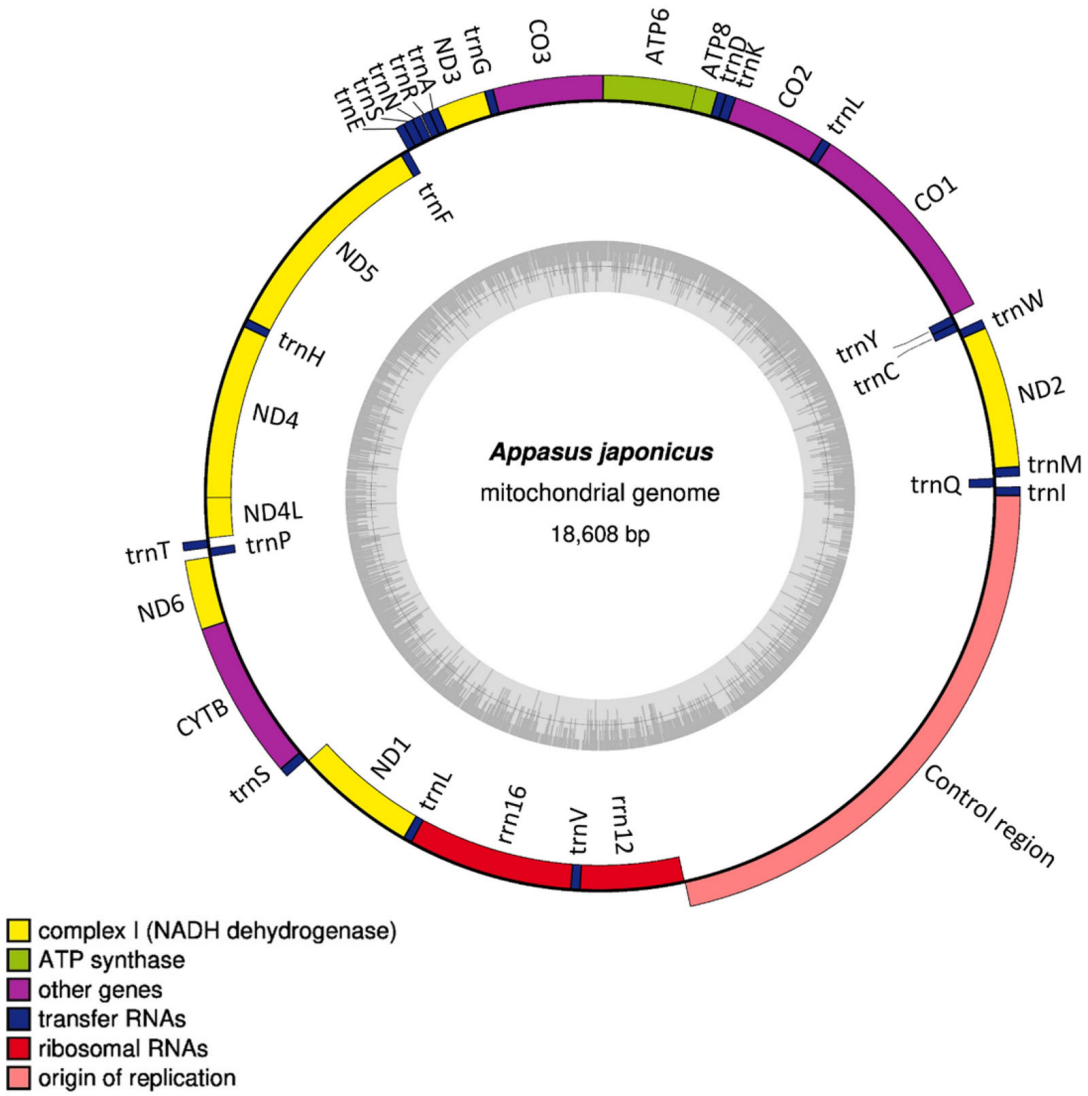
Gene map of the *Appasus japonicus* mitochondrial genome. Genes inside the circle are transcribed clockwise, and genes outside are transcribed counter-clockwise. The dark grey inner circle corresponds to the GC content, and the light-grey circle corresponds to the AT content.

The assembled mitogenome of *A. japonicus* (GenBank accession No: OK012605) showed a length of 18,608 bp and an overall GC content of 29.4%, along with a total nucleotide composition of A − 41.9%, C − 17.5%, G − 11.9%, and T − 28.7%. The mitogenome consists of 13 protein-coding genes (PCGs), 22 tRNA genes, and two ribosomal RNA (rRNA) genes (Figure 3). The genome structure, gene order, and total gene number of the *A. japonicus* mt genome are identical to those in *D. rusticus* (FJ456940), which is a closely related genus. The phylogenetic tree revealed that *A. japonicus* distributed in the clade containing the species *D. rusticus* and the relationships was supported by a high bootstrap value (BS = 100) (Figure 2).

## Conclusion

The mitochondrial genome of *A. japonicus* was 18,608 bp and contained thirteen protein-coding genes, 22 transfer RNAs, two ribosomal RNAs and an AT-rich region. A phylogenetic analysis of 21 complete mitochondrial genomes of the registered Order Hemiptera suggested that *D. rusticus* was most closely related to *A. japonicus*. Also, *A. japonicus* and the genus *Lethocerus* formed a monophyletic group. We expect that the present results will facilitate further investigations into the phylogenetic relationships in the Belostomatidae.

## Data Availability

The genome sequence data that support the findings of this study are openly available in GenBank of NCBI at https://www.ncbi.nlm.nih.gov. under the accession no. OK012605. The associated BioProject, SRA, and Bio-Sample numbers are PRJNA753318, SRR15404221 and SAMN20693876, respectively.
